# Supplementary figures and images for: Parental breeding age effects on descendants’ longevity interact over 2 generations in matrilines and patrilines
Source: PLoS Biol. 2019 Nov 25;17(11):e3000556. doi: 10.1371/journal.pbio.3000556 (PMC6901263; doi:10.1371/journal.pbio.3000556)

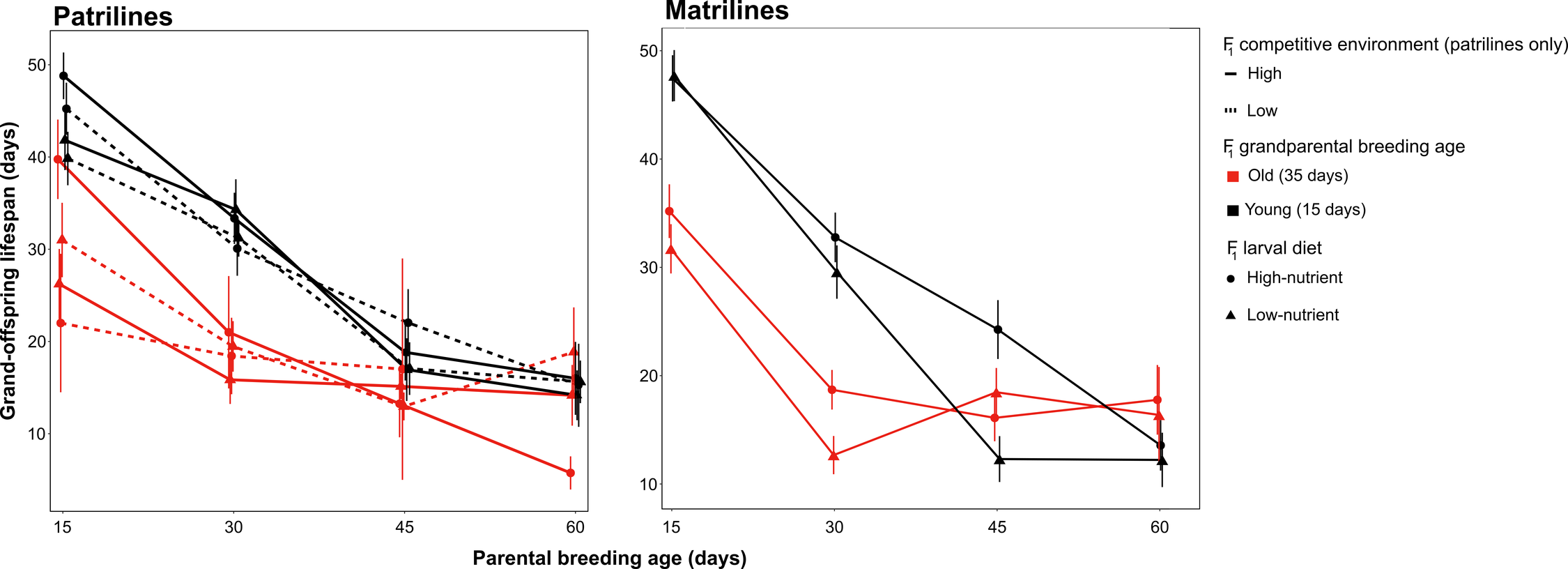

Supplement: S1 Fig — Black lines represent F3 individuals descended from F1 males and females bred at a young age (15 days old) and red lines signify individuals descended from old (35 days old) grandparents. In patrilines only, individuals descended from F1 males that were subjected to either a high or low competitive environment are represented by a solid or dotted line, respectively. F3 grand-offspring of F1 grandparents reared on a high-nutrient larval diet are represented by a circle, and low-nutrient larval diest is represented by a triangle. All points represent means. Bars represent SEM. Underlying data can be found in the Dryad Repository: https://doi.org/10.5061/dryad.2rbnzs7hw. F1, grand-parental generation; F2, female and male offspring; F3, grand-offspring. (TIF) [file pbio.3000556.s001.tif]

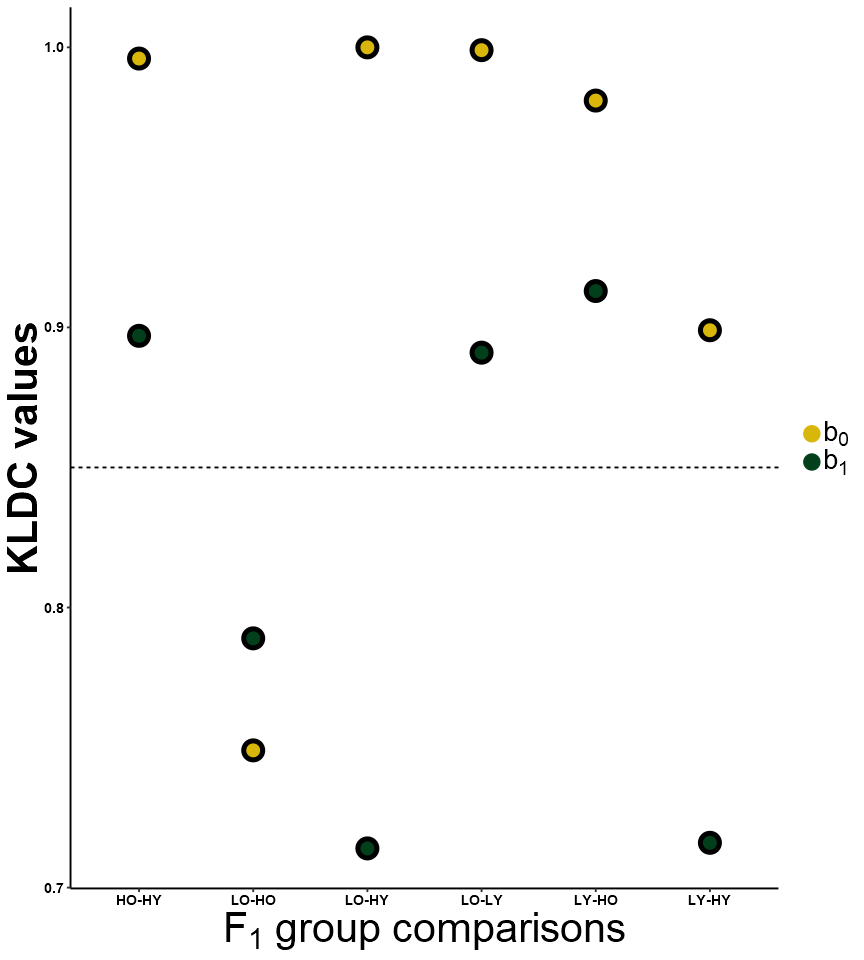

Supplement: S2 Fig — Underlying data can be found in the Dryad Repository: https://doi.org/10.5061/dryad.2rbnzs7hw. HO, High Nutrient Old Breeding treatment; HY, High Nutrient Young Breeding treatment; KLDC, Kullback-Leibler discrepancy calibration; LO, Low Nutrient Old Breeding treatment; LY, Low Nutrient Young Breeding treatment. (TIF) [file pbio.3000556.s002.tif]

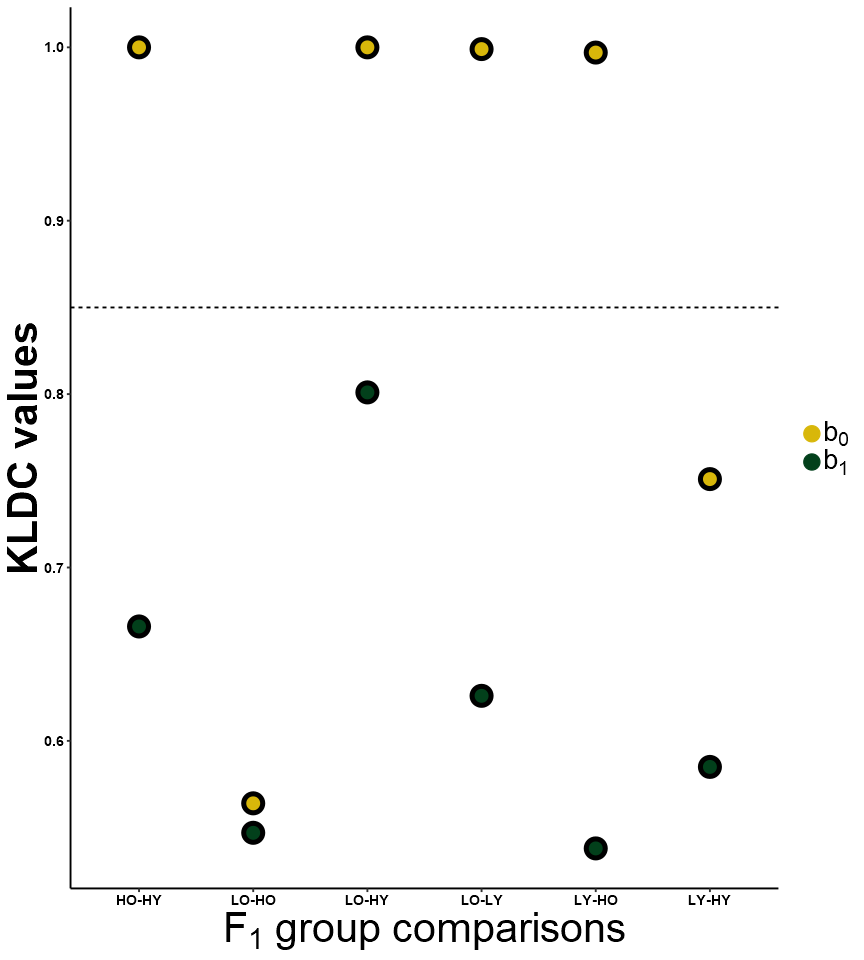

Supplement: S3 Fig — Underlying data can be found in the Dryad Repository: https://doi.org/10.5061/dryad.2rbnzs7hw. HO, High Nutrient Old Breeding treatment; HY, High Nutrient Young Breeding treatment; KLDC, Kullback-Leibler discrepancy calibration; LO, Low Nutrient Old Breeding treatment; LY, Low Nutrient Young Breeding treatment. (TIF) [file pbio.3000556.s003.tif]

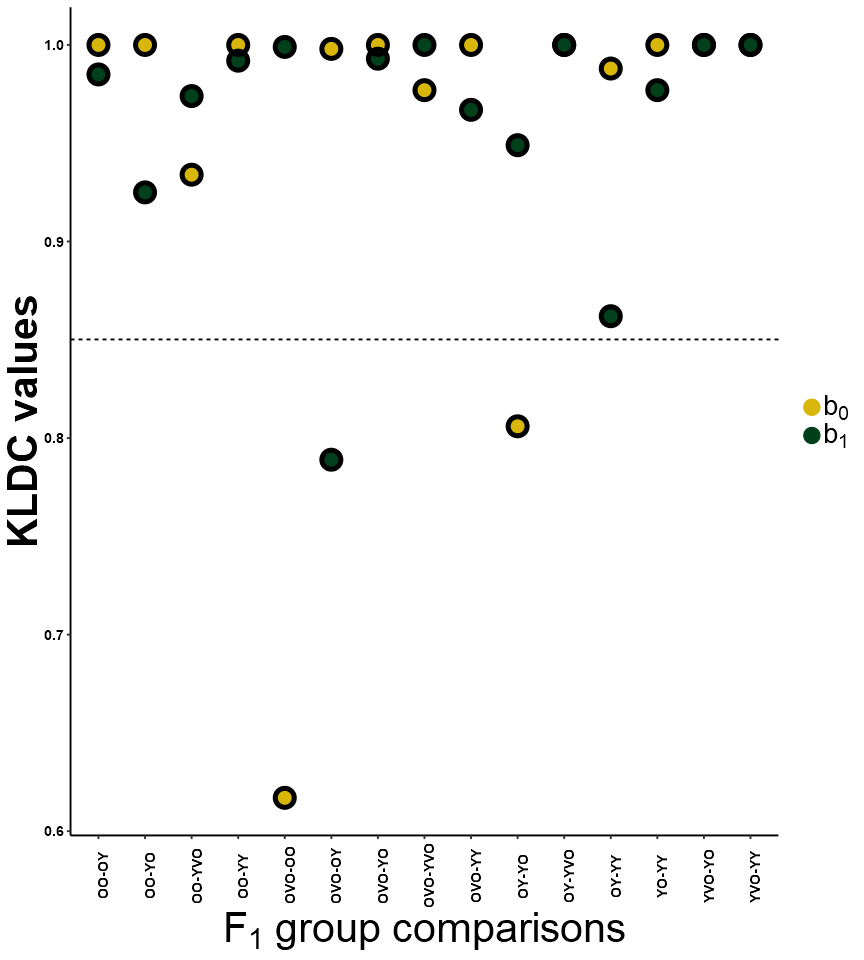

Supplement: S4 Fig — Underlying data can be found in the Dryad Repository: https://doi.org/10.5061/dryad.2rbnzs7hw. F1, grand-parental generation; F2, female and male offspring; KLDC, Kullback-Leibler discrepancy calibration; OO, Old F1 breeding age Old F2 breeding age; OY, Old F1 breeding age Young F2 breeding age; YO, Young F1 breeding age Old F2 breeding age treatment; YVO, Young F1 breeding age Very old F2 breeding age; YY, Young F1 breeding age Young F2 breeding age. (TIF) [file pbio.3000556.s004.tif]

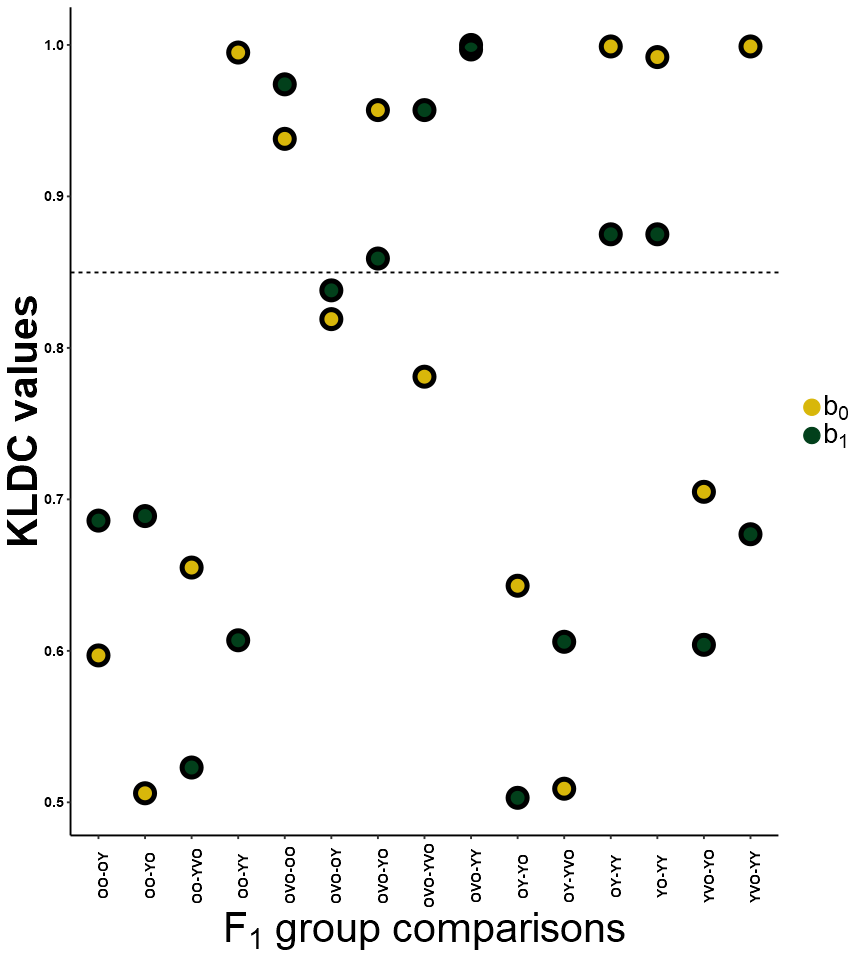

Supplement: S5 Fig — Underlying data can be found in the Dryad Repository: https://doi.org/10.5061/dryad.2rbnzs7hw. F1, grand-parental generation; F2, female and male offspring; KLDC, Kullback-Leibler discrepancy calibration; OO, Old F1 breeding age Old F2 breeding age; OY, Old F1 breeding age Young F2 breeding age; YO, Young F1 breeding age Old F2 breeding age treatment; YVO, Young F1 breeding age Very old F2 breeding age; YY, Young F1 breeding age Young F2 breeding age. (TIF) [file pbio.3000556.s005.tif]

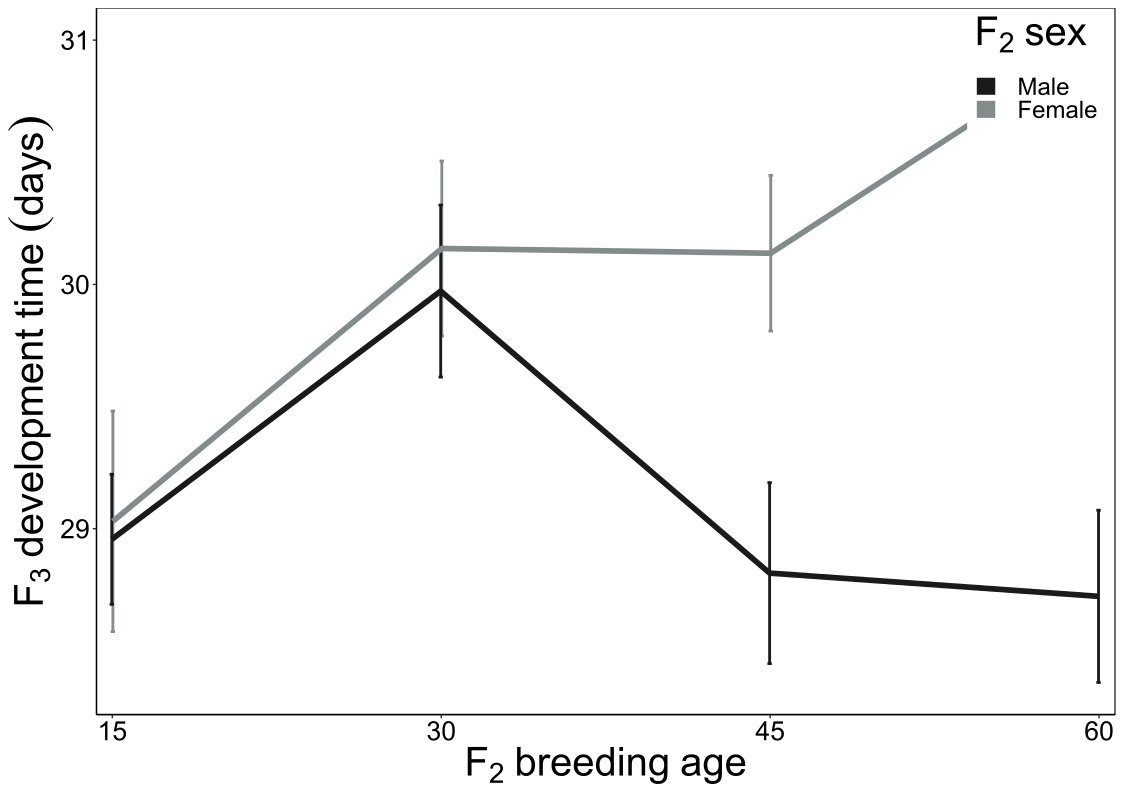

Supplement: S6 Fig — Solid grey lines represent F3 individuals descended from F2 females and solid black lines represent F3 individuals descended from F2 males. Bars represent SEM. Underlying data can be found in the Dryad Repository: https://doi.org/10.5061/dryad.2rbnzs7hw. F2, female and male offspring; F3, grand-offspring. (TIF) [file pbio.3000556.s006.tif]

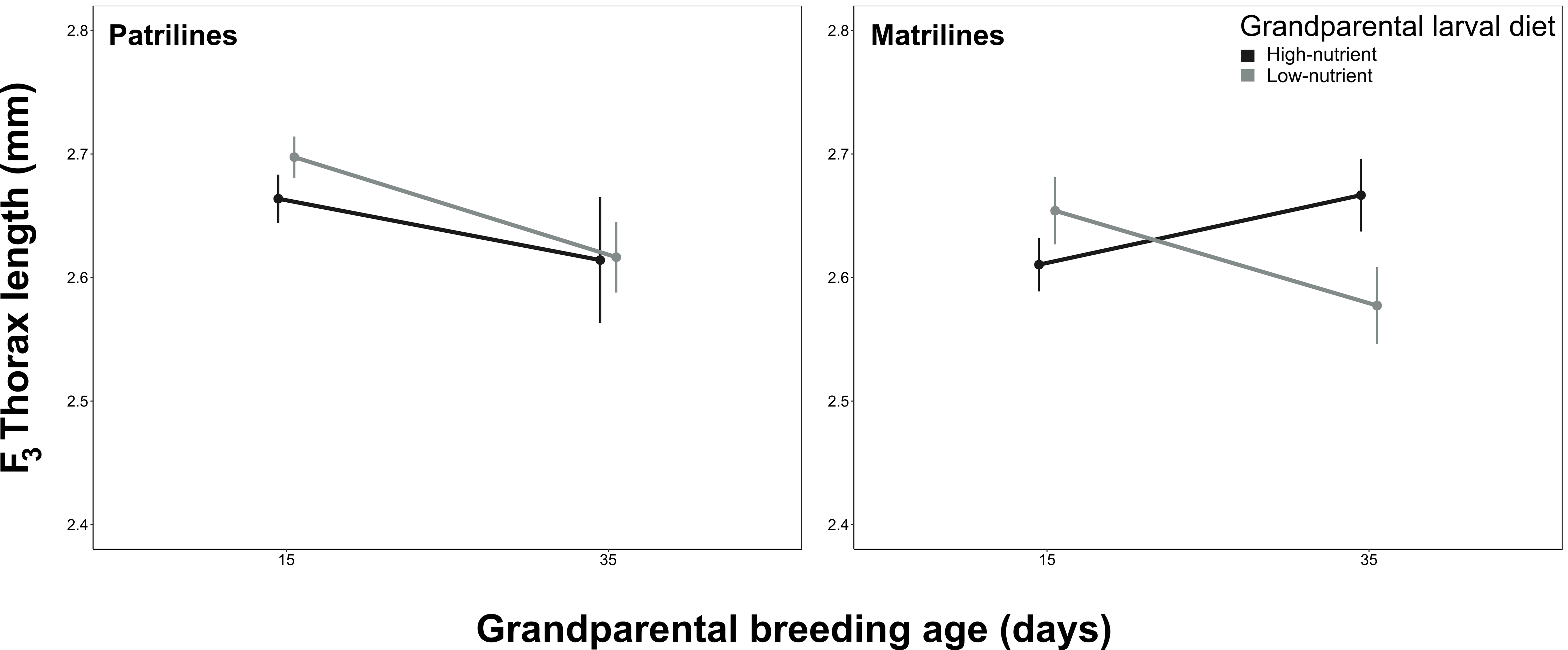

Supplement: S7 Fig — Solid grey lines represent effects of F1 individuals reared on reared on a poor larval diet, and solid black lines represent the effects of F1 individuals reared on a rich larval diet. Bars represent SEM. Underlying data can be found in the Dryad Repository: https://doi.org/10.5061/dryad.2rbnzs7hw. F1, grand-parental generation; F3, grand-offspring. (TIF) [file pbio.3000556.s007.tif]
