# Supplementary material for: Parental breeding age effects on descendants’ longevity interact over 2 generations in matrilines and patrilines
Source: PLoS Biol. 2019 Nov 25;17(11):e3000556. doi: 10.1371/journal.pbio.3000556 (PMC6901263; doi:10.1371/journal.pbio.3000556)
Supplement: S1 Text — F3, grand-offspring (DOCX) [file pbio.3000556.s023.docx]

## Parental breeding age effects on descendants’ longevity interact over two generations in matrilines and patrilines.

**Supplementary information**

## EFFECTS ON BODY SIZE AND DEVELOPMENT TIME

In both matrilines and patrilines we observed significant effects of F_3_ sex on F_3_ body size (Table S13). In the matrilineal dataset however, an interaction between F_1_ larval diet and F_1_ age was also observed, such that grand-mothers reared on a poor larval diet and bred at a ‘young’ age produced larger grand-offspring. In the patrilineal dataset we also detected an interaction of F_2_ age and F_2_ sex (Table S13), such that F_3_ body size increased as a mothers’ breeding age increased, whereas F_3_ offspring that descended from older fathers showed a significant decrease in body size (Fig. S6).

For F_3_ development time we did not detect any significant effects apart from a marginally significant interaction between F_2_ sex and F_2_ age within patrilines (Table S14) suggesting that paternal breeding age has a direct influence on offspring developmental trajectory. Offspring descended from old fathers had a much shorter development time than offspring descended from old mothers (Fig. S6).

Previous work on *T. angusticollis* has shown that macronutrients in the maternal and paternal larval diets have complex effects on a number of offspring traits, including body size, juvenile viability and development time [1–3]. Here, we observed an interaction of grand-maternal age and larval diet on grand-offspring body size in matrilines, where grand-offspring body size decreased with grand-maternal age at breeding when grandmothers were reared on low-nutrient diet but increased with grand-maternal age at breeding when grandmothers were reared on high-nutrient larval diet (Fig.S4). In patrilines, we observed F_2_ sex × F_2_ age and F_1_ age × F_3_ sex interactions for body size, indicating that body size is negatively affected by increased parental and grandparental breeding age, but this effect varies as a function of parental and offspring sex (Fig. S5). We also detected an F_2_ sex × F_2_ age interaction effect on development time (Fig. S6), indicating that offspring development time increased with maternal age at breeding but decreased with paternal age at breeding. A number of studies on other species have also found that maternal age at reproduction can affect offspring body size, and theory suggests that such patterns could reflect adaptive maternal effects [4]. However, the effects are often complex and difficult to interpret. For example, in earth mites, younger mothers produce offspring of a smaller body size (and thus lower fitness), but older mothers produce larger and better provisioned offspring [5].

**References**

1. Bonduriansky R, Head M. Maternal and paternal condition effects on offspring phenotype in Telostylinus angusticollis (Diptera: Neriidae). J Evol Biol. 2007;20: 2379–2388. doi:10.1111/j.1420-9101.2007.01419.x

2. Bonduriansky R, Runagall-mcnaull A, Crean AJ. The nutritional geometry of parental effects : maternal and paternal macronutrient consumption and offspring phenotype in a neriid fl y. Funct Ecol. 2016; doi:10.1111/1365-2435.12643

3. Hooper AK, Spagopoulou F, Wylde Z, Maklakov AA, Bonduriansky R. Ontogenetic timing as a condition-dependent life history trait: High-condition males develop quickly, peak early and age fast. Evolution (N Y). 2017; 1–15. doi:10.1111/evo.13172

4. Kindsvater HK, Rosenthal GG, Alonzo SH. Survival costs of reproduction predict age-dependent variation in maternal investment. J Evol Biol. 2011;24: 2230–2240.

5. Plaistow SJ, Clair JJHS, Grant J, Benton TG. How to Put All Your Eggs in One Basket : Empirical Patterns of Offspring Provisioning throughout a Mother ’ s Lifetime. Am Nat. 2007;170: 520–529. doi:10.1086/521238
