# Supplementary material for: Parental breeding age effects on descendants’ longevity interact over 2 generations in matrilines and patrilines
Source: PLoS Biol. 2019 Nov 25;17(11):e3000556. doi: 10.1371/journal.pbio.3000556 (PMC6901263; doi:10.1371/journal.pbio.3000556)
Supplement: Attachment — Submitted filename: Wylde et al_2019_response_R.1_ZW.docx [file pbio.3000556.s024.docx]

September #, 2019

**Re: Parental breeding age effects on descendants’ longevity interact over two generations in matrilines and patrilines**.

Dear Editor,

We appreciate the opportunity to revise our manuscript, ‘Parental breeding age effects on descendants’ longevity interact over two generations in matrilines and patrilines.’ We value the thoughtful and constructive suggestions made by the reviewers and it is our belief that the manuscript has been substantially improved as a result.

Following this letter are the reviewer comments with our responses in blue italics, including how and where the text or figure was modified. The revision has been developed in consultation with all co-authors and each has given approval to this revision.

We look forward to hearing from you soon.

Thank you,

Zac Wylde (on behalf of all authors)

University of New South Wales, Sydney, Australia

**Reviewer #1**: Wylde and co-authors present a well-motivated and thorough examination of parental age effects in longevity in neriid flies. The authors do an excellent job of placing their study in broader context, and pointing to various avenues of further work that might help to clarify the role of parental age in the evolution of ageing, as well as the potential mechanisms. These issues are currently neglected and I think the authors' contribution will be a strong addition to the ageing literature. The statistical analyses are certainly sufficient, although presentation / interpretation of the lme4 models need a little work (detailed below). My comments are therefore fairly minor, centred mostly around clarifying a few aspects, improving presentation of results, and a suggestion for ending on a stronger note than the current manuscript.

79-82: consider dropping “we investigated” / “we investigated and compared” from each bullet point, particularly as “we addressed” is in the opening clause. This redundancy makes this part long and difficult to digest when it should be a punchy section about the authors’ aims.

*We have amended this repetition by changing our choice of verbs to “examined”, “comparing” & “analysis.” See lines 90-94.*

92-94: a note of lifespan, patterns of reproductive senescence etc from previous studies of these flies would be useful here to show the reader why these ages have been chosen.

*We have added in a reference and reasoning for our choice of breeding ages, see lines 164-168.*

Table 1: For categorical variables, we need to know what the reference levels / contrasts are, else having a table of prameter estimates isn’t particularly meaningful. I’m also wondering why the authors use likelihood ratio tests to assess significance of covariates, but then use p-values from F-tests in their table to illustrate significant effects? It seems to me that a more coherent setup would be to include estimate, SE and F-statistic for each term in the table, then include chi-square statistic and associated p-value for interactions / main effects that are not involved in interactions. For an example, see Table 2 of Buser et al 2013 Fun Ecol (DOI: 10.1111/1365-2435.12188).

*Table 1 has been amended to include F-statistics, as well as Χ^2^ and P values based on likelihood ratio tests, as suggested by the reviewer. The results are unchanged from the original version.*

292-302: Please clarify the abbreviations for treatment groups somewhere in here. Also, consider including the confidence intervals for each estimate (or at least noting that the posterior distributions are shown in Fig 4). While on the subject, I’m not sure the authors discuss the fact that high-nutrient old age groups seem to have much higher variance in both survival and mortality than all other groups. Is there any reason that that might be the case?

*Lines 342-346: we have added definitions of abbreviations in brackets. Line 353-359: we have clarified in the Fig. 4 caption that the plots show posterior distributions of the parameters with 95% CIs.*

*The larger variances for lifespan and mortality rate of descendants of old-breeding F1 males reflect smaller sample sizes resulting from mortality before 35 days of age. We have noted this in Results (lines 380-382).*

Figure 4: The figure text (axes labels etc) is barely readable despite being a full page – please enlarge this. Furthermore, the legend doesn’t adequately describe the figure panels – this needs to be clear to the reader that the left hand side shows effect sizes and (95%?) posterior distributions for the 4 treatment groups, and that the right hand side shows how these estimates translate to survival and mortality rates over time. In addition, the x axis states that age is in years – I assume this is a typo, otherwise I must congratulate the authors even more on the completion of this study!

*Figures 4 & 5 axes labels etc have been enlarged and the legend has been expanded to clarify what the panels show. “Years” was indeed a typo, and has been changed to “Days.”*

344-348: I don’t quite get how the ‘indeed’ clause follows what comes before it. Is there a step in the logic missing here? Or it just needs to be rephrased somehow to make it clear what the authors mean.

*Line 405. Changed to “Previous studies of T. angusticollis showed that….’*

443-452: I think the authors do themselves a little bit of a disservice with their final paragraph, and could make a stronger case for the usefulness of their results in the broader picture. Currently it feels like the paper fades to ‘something was seen, and more work should be done’. This might even just be a case of rewriting the first sentence in active voice rather than passive, but I think the last line could also be reworked to make the case that the authors have indeed contributed results that demonstrate the need for further work in a neglected aspect of the evolution of ageing.

*Thanks for this suggestion. We have edited the closing paragraph to more clearly emphasize the contribution that this study makes (lines 543-557).*

**Reviewer #2**: This MS investigates the impact of age on aging in offspring and grand-offspring in a fly. It's major advance is that it comprehensively investigates both male and female age on the aging of descendants. They find that male age has at least as big an impact as female age, both at the parental and grandparental stage, and the resulting lifespan change in descendants is very impressive (up to 40%). In my view, this paper is a substantial advance in the field, and will become a citation classic. So I am happy to recommend it for publication. It is very well written and comprehensively covers the literature, and I have only very minor suggestions for improvement.

Minor issues:

The authors briefly discuss the impact of females potentially investing less in the offspring of older males, but they do not mention the possibility that mating experience (by both males and females) correlates with age in their design. This also might change investment by F1 and F2 flies in ejaculates and eggs. This is not really a flaw in their design, but I think it is worth a sentence or two.

*This is a valid point. We have added some discussion of the potential effects of mating experience on male ejaculate traits and female fecundity (lines 511-522).*

Figure 1 doesn't seem to reflect the design. My understanding is that the 15 and 35 day old broods from the F1 became the F2, who were reared under standard conditions and mated at age 15, 30, 45 and 60. The current diagram does not depict that. I cannot see why the F2 flies have a branching arrow beneath them- what are the F3 flies being split into two groups for? Instead, a simple single arrow point down from each of the F2 male/female symbols would make more sense (ie twice as many arrows as currently used, but none of them split at the end).

*We have modified Figure 1 to improve clarity and show sample sizes for each treatment combination.*

**Reviewer #3**: This manuscript reports the results of an experiment testing the possible effect of grandmaternal and grandpaternal age on offspring lifespan. The article is well written and reports interesting results. However, I have a few major criticisms.

The first point is that, by reading the manuscript, one might misleadingly think that paternal age effects on the progeny have not been studied, yet. This is not true since during the last years several papers have focused on paternal age and have already reported negative effects on offspring lifespan and/or LRS (see for instance Schroeder et al. 2015 PNAS). I think that this should be fully acknowledged.

*We acknowledge in our Introduction (e.g. first paragraph) that paternal age effects have been reported in previous studies. The Schroeder et al. 2015* *paper was originally referenced in our paper but somehow got deleted in the submitted version. This paper is now referenced at line 24 and discussed in line 28.*

I found the experimental design unnecessary complex. Looking at how the environment modulates grandparental effects on offspring longevity is of course interesting, but to me the first step should have been to make sure that such grandparental effects exist, using a more straightforward experimental design. The choice of the environmental traits that have been manipulated is also questionable, especially male’s competitive environment, given that previous work showed no effect of male combat on reproductive aging in this species.

*Male combat did not affect male longevity and did not have a clear effect on several measures of male reproductive ageing (see Hooper et al. 2017. Evolution 71: 671-685). However, the current study is the first to test whether male combat modulates male breeding-age effects on descendants’ longevity. We did not know whether male combat would have such effects when designing this study. Given how little is known about the potential for environmental factors to modulate parental age effects, we feel that an investigation of the potential role of male combat is interesting and worthwhile.*

The choice of the age at breeding for the different generations should also be better explained and justified. Grandparents were let to breed at the age of 15 and 35 days, but I did not find any justification of these ages and why 15 day old flies are young and 35 day old flies are old. This point is crucial to me especially because at the following generation flies were bred at the age of 15, 30, 45 and 60 days. Therefore, 35 day old flies seem to be middle-aged rather than old. I think the authors should provide data on the onset of reproductive aging, as to justify that 35 day old flies are indeed “old”. The other concern is that males and females were bred at the same age but there is evidence showing that the onset and rate of aging might differ between sexes. Is the onset of reproductive aging similar between sexes in this species? If not the comparison of grandmaternal and grandpaternal effects on offspring longevity might be flawed.

*We use “young” vs. “old” simply to distinguish earlier vs. later ages at breeding. Given that both life expectancy and ageing rate differ between captive and natural populations in this species (Kawasaki et al. 2008. American Naturalist 172: 346-357), it’s not clear whether it’s most appropriate to refer to a 35 day old fly as “old” or “middle-aged.” In any case, 20 days is a very long time in the life of a fly, and we show that this difference in age at breeding has dramatic consequences for descendants’ longevity.*

*We chose to contrast effects of F1 breeding at 15 days of age (which corresponds to the prime breeding age, shortly after the typical age of full reproductive maturity in T. angusticollis) with effects of breeding at 35 days of age (which corresponds to the age at which mortality begins to increase rapidly in T. angusticollis males, while probability of mating and ability to stimulate females to oviposit begin to decline; see Hooper et al. 2017. Evolution 71: 671-685). In the F_2_ generation, we investigated similar ages at breeding but also extended the design to investigate effects of breeding at even older ages.*

*We do not have equally detailed data on reproductive ageing in females, but mortality rate begins to increase rapidly at similar ages in the sexes (Kawasaki et al. 2008. American Naturalist 172: 346-357) and oviposition rate declines rapidly after age 30 days in females (unpublished data). The available data therefore suggest that the sexes age at similar rates in this species. However, we agree with the reviewer that more detailed data on rates of reproductive ageing in females vs. males would be valuable and would help to calibrate the relative magnitudes of male vs female breeding age effects reported in this study. We have added some further justification for our choice of breeding ages (lines 164-170), and some discussion of the potential effects of F_3_ housing (lines 524-531).*

The final point related to the experimental design is that focal flies (the F3 generation) were maintained in isolation and therefore could not breed. This is of course a very artificial condition, very different from what these animals experience in the wild. This brings me to the point of the ecological relevance of the experiment. As also acknowledged by the authors in the discussion, flies have a much longer longevity in the lab compared to the wild, therefore we can reasonably question whether the results reported here have any ecological relevance.

*Our findings are likely to have ecological relevance because neriid flies can live for weeks in the wild (Kawasaki et al. 2008. American Naturalist 172: 346-357). However, we agree with the reviewer that the magnitude of the effects that we observed is difficult to gauge for natural populations, given differences in longevity and ageing rate between captive and wild flies, and we acknowledge this uncertainty in the Discussion (lines 417-429). We certainly agree that more work is needed to determine the importance of breeding age effects in natural populations. We do note, however, that data on longevity and ageing in natural populations of insects are extremely scarce (neriid flies being one of the few species for which any such data are available). The ecological interpretation of all laboratory studies of longevity and ageing in insects is therefore similarly uncertain.*

*We agree with the reviewer that housing F3 flies in isolation likely affected their longevity. We do not know whether effects of housing in isolation vs. in groups would interact with effects of parental and grandparental ages at breeding. These are interesting questions for future investigation, but they are beyond the scope of the present study. We note that individual housing is likely to have an effect in the Discussion (lines 524-531).*

Statistical analyses

I did not find any mention of the sample size, except the overall number of male and female flies per generation reported in figure 1. Looking at figure 2, it seems that much fewer F3 flies were available in the F1 high nutrient diet x old breeding age group. Why is this so? I would recommend reporting more precisely the number of flies per group.

*Sample sizes for group combinations in Figure 2 have been placed above each violin. Samples for each grouping are provided now in Figure 1. As we noted above, high-nutrient diet F1 flies experienced a higher mortality rate, and fewer focal individuals therefore survived to breed at 35 days. We have noted this in Results (lines 380-382).*

Table 1. I do not understand if this table reports the results of the full model or if there have been a model selection procedure. If not, I think this should be done because many of the effects (especially the interaction terms) seem to be borderline (p values between 0.01 and 0.05). I would also recommend providing an estimate of effect size.

*We selected a model structure that corresponded to our experiment design, and simplified the patriline model by removing the “social environment” treatment (which had no effects) in order to obtain comparable models for patrilines and matrilines (see lines 229-239). We did not simplify our models beyond this so as to preserve model structures that accurately reflected our experiment design. Table 1 shows results for the final model. In the supplement, we also show models that include development time and body size to determine whether these covariates mediated treatment effects on lifespan. Marginal effect sizes (R^2^), calculated using the method developed by Nakagawa & Shielzeth (2013), have been added to Table 1 and explained in methods (line 251-254).*

Lifespan was analysed using a normal distribution of errors, but very often longevity is right skewed with a few individuals having extreme lifespan. Did you check the assumptions underlying the use of LMM?

*Homoscedasticity of error was checked and found to be normal for both patriline and matriline lifespan data sets.*

*Distribution of errors for matriline data.*

*Distribution of errors for patriline data.*

Figure 2. This might be a matter of taste, but I do not think “violins” are particularly useful here. I am also wondering why you decided to split the data according to F1 breeding age and diet, given that diet is never significant. Instead, I would recommend illustrating how good was your model to fit the data.

*We feel that the violin plots are informative by illustrating the probability densities of where the bulk of the variation is located. The reason we show the data split by F1 diet is to highlight the fact that it has little effect on F3 lifespan when compared to F1 breeding age. We feel that it’s important to illustrate not only the significant effects, but also the nonsignificant ones. Conditional whole-model R^2^ values have been added to the caption for Table 1.*

Figure 3. Sample sizes should be reported here. How many flies were in the group 35 day old F1 x 60 day old F2.

*Sample sizes for all treatment combinations are now shown in Figure 1.*

**Reviewer #4**: Wylde et al., ‘Parental breeding age effects on descendants’ longevity interact over two generations in matrilines and patrilines’, PBIOLOGY-D-19-02204 

This is a potentially important paper assessing parental and grand-parental breeding age effects on lifespan in the neriid fly Telostylinus angusticollis. The study not only takes parental and grand-parental age effects along the patriline and matriline into account but also looks at interactive - potentially cumulative - effects across generations and addresses whether inter-/transgenerational age effects are sensitive to environmental modifiers (diet, male competitive behaviors). 

The key findings reported are that older breeding ages along the patriline and matriline are associated with negative effects on lifespan in offspring (which is in line with findings in other species) and the authors also provide some evidence for cumulative effects of advanced grand-parental and parental ages on lifespan in progeny (this aspect has not been addressed by prior studies). Parental/grand-parental age effects were not modified by the environmental factors studied. 

Overall, the study appears to be well-designed, takes an extensive approach considering a number of possible parental/grand-parental factors and may represent an important contribution to the existing literature on multi-generational age effects. 

This reviewer has the following specific comments on the manuscript:

1)      It would be important to specify for each of the different breeding age groups what proportion of animals in the F1/F2 population died prior to reaching the assigned breeding age. Also, do the authors have data regarding possible age-related changes in breeding success? These aspects are important because of selection processes that are expected to affect the various breeder age groups in different ways.

*Figure 1 now shows sample sizes for each treatment combination. Mortality rate was higher for flies (especially males) reared on nutrient-rich larval diet, and fewer focal individuals reared on nutrient-rich diet therefore survived to breed at older ages. We note this in Results (lines 380-382), and discuss potential selection effects associated with this differential mortality (lines 532-542). Although we cannot exclude selection effects, we feel that such effects are unlikely to contribute substantially to our results because descendants of older individuals exhibited lower life expectancies.*

*Many aspects of reproductive performance decline at older ages in males (Hooper et al. 2017. Evolution 71: 671-685) and females (unpublished data). However, further work is needed to determine how age-related changes in aspects of reproductive performance (e.g. male seminal fluid quality vs. sperm quality, female egg quality, etc) might contribute to the breeding age effects that we report.*

2)      Please clarify how lifespans measured in the present study compare to published lifespan data in Telostylinus angusticollis.

*Lines 164-168 outline how we chose the ages to test for this study. We compare lifespans measured in the current study with estimates from previous studies in the revised Results (lines 314-320).*

3)      Page 7, lines 145-165: The same F1/F2 animals were used at different ages to generate offspring of the various breeder age groups. Is it correct that, as a consequence, young breeder offspring were always generated by naïve animals and older breeder offspring were always generated by experienced breeders? In that case, it would be essential to make sure that indeed breeder age is the critical experimental variable (and not parity/prior breeding experience). Do the authors have offspring lifespan data derived from naïve breeders of the various parental/grand-parental age groups?

*As the reviewer notes, we cannot separate potential effects mating experience from the effects of age in our analysis. Effects of mating experience on descendants’ longevity have not been investigated in T. angusticollis and, to our knowledge, such effects have not been reported in other species. However, we have added some discussion of the potential for such effects (lines 511-522).*

4)      Fig. 1: It is essential to spell out how many animals precisely were used within all the different experimental groups covered by the study design and how many breeders they were derived from. The scheme in Fig. 1 should be modified to represent a tree showing all experimental groups. All possible combinations of factors examined and the number of associated animals and breeders should be presented within this scheme.

*Figure 1 now shows sample sizes for all treatment combinations.*

5)      Fig. 2 and Fig. 3: These figures show data that were stratified by some of the factors used in the present study but were collapsed across others. While it may be useful to simplify data presentation in the main figures, it would still be informative to provide additional figures - within the Supplementary Material - wherein the data presented in Figs. 2 and 3 are stratified by all the relevant factors that went into the analysis (sex, diet, competition etc., as applicable).

*We have added Figure S1 showing plots of the data stratified by F1 diet, age, competition environment, and F2 ages.*

6)      For all measures examined (lifespan, thorax length etc.), please provide tables specifying key data distribution metrics (mean, standard deviation; also, number of animals, number of breeders) for all the different groups/combination of factors (see also point 4 above). This would be an essential addition and could be placed in the Supplementary Material.

*We have added Table S1 showing summary statistics. Sample sizes are shown in Figure 1.*

7)      Please check reference 37 (Sharma et al., 2016) – it does not appear to support the statement on page 4, lines 57-59 of the manuscript.

*Thank you for picking up on this reference manager error. We have corrected this in the revised manuscript.*
